# Spontaneous rotational symmetry breaking in KTaO_3_ heterointerface superconductors

**DOI:** 10.1038/s41467-023-38759-0

**Published:** 2023-05-26

**Authors:** Guanqun Zhang, Lijie Wang, Jinghui Wang, Guoan Li, Guangyi Huang, Guang Yang, Huanyi Xue, Zhongfeng Ning, Yueshen Wu, Jin-Peng Xu, Yanru Song, Zhenghua An, Changlin Zheng, Jie Shen, Jun Li, Yan Chen, Wei Li

**Affiliations:** 1 State Key Laboratory of Surface Physics and Department of Physics, Fudan University, Shanghai, 200433 China; 2 ShanghaiTech Laboratory for Topological Physics & School of Physical Science and Technology, ShanghaiTech University, Shanghai, 201210 China; 3 Beijing National Laboratory for Condensed Matter Physics and Institute of Physics, Chinese Academy of Sciences, Beijing, 100190 China; 4 ShanghaiTech Quantum Device Lab, ShanghaiTech University, Shanghai, 201210 China; 5 Institute for Nanoelectronic Devices and Quantum Computing, Fudan University, Shanghai, 200433 China; 6 Songshan Lake Materials Laboratory, Dongguan, 523808 China

**Keywords:** Surfaces, interfaces and thin films, Superconducting properties and materials

## Abstract

Broken symmetries play a fundamental role in superconductivity and influence many of its properties in a profound way. Understanding these symmetry breaking states is essential to elucidate the various exotic quantum behaviors in non-trivial superconductors. Here, we report an experimental observation of spontaneous rotational symmetry breaking of superconductivity at the heterointerface of amorphous (a)-YAlO_3_/KTaO_3_(111) with a superconducting transition temperature of 1.86 K. Both the magnetoresistance and superconducting critical field in an in-plane field manifest striking twofold symmetric oscillations deep inside the superconducting state, whereas the anisotropy vanishes in the normal state, demonstrating that it is an intrinsic property of the superconducting phase. We attribute this behavior to the mixed-parity superconducting state, which is an admixture of *s*-wave and *p*-wave pairing components induced by strong spin-orbit coupling inherent to inversion symmetry breaking at the heterointerface of a-YAlO_3_/KTaO_3_. Our work suggests an unconventional nature of the underlying pairing interaction in the KTaO_3_ heterointerface superconductors, and brings a new broad of perspective on understanding non-trivial superconducting properties at the artificial heterointerfaces.

## Abstract (web summary)

Superconducting interfaces involving KTaO3 have recently attracted attention due to their relatively high transition temperature. Here, the authors study amorphous-YAlO_3_/KTaO_3_ interfaces and find two-fold symmetry in the superconducting regime, possibly due to a mixed-parity superconducting state.

## Introduction

The study of heterointerface superconductivity has been a central theme in condensed matter physics communities^1^. Due to the presence of inversion symmetry breaking and the particular interactions found at their interface between two constitute materials, the strong interplay between the electrons with Coulomb interaction and the interfacial electron-phonon coupling gives rise to novel superconducting behaviors, providing an ideal platform for understanding the underlying rich physical properties and developing the next-generation quantum technologies^2–4^. The archetypal heterointerface superconductivity has been experimentally observed at the heterointerface of crystalline (c)-LaAlO_3_/SrTiO_3_ with a superconducting transition temperature (*T*_*c*_) of 250 mK^5^, which ignites the first fire in heterointerface superconductivity research. Strikingly, a variety of appealing quantum phenomena has also been revealed at the superconducting SrTiO_3_ heterointerfaces, such as the coexistence of ferromagnetism and superconductivity^6–8^ and the gate-tunable superconductivity^9–13^, indicative of a possible unconventional and non-trivial superconducting phase as the ground state^14^. Unfortunately, the extremely low *T*_*c*_ of SrTiO_3_ heterointerface superconductors is a critical challenge, preventing extensive attentions to further unveil the origin of these emergent quantum phases.

Very recently, unexpected superconductivity is experimentally observed at the heterointerface between polycrystalline (p)-EuO [or amorphous (a)-LaAlO_3_] and KTaO_3_ single-crystal substrates which shows a *T*_*c*_ ~ 2 K^15,16^, approximately one order of magnitude higher than that of c-LaAlO_3_/SrTiO_3_^5^, evoking an exciting opportunity to study the physical properties of heterointerface superconductivity. Although KTaO_3_ shares many common features with SrTiO_3_^15–18^, the superconductivity of KTaO_3_ heterointerfaces behaves in a quite different manner. Remarkably, the superconductivity of these heterointerfaces exhibits a strong dependence on the KTaO_3_ crystalline orientations by compared to the SrTiO_3_ crystalline orientation independence of superconductivity^19–24^. Furthermore, considering the fact that the strong spin-orbit coupling associated with the heavy Ta in 5*d* orbitals of KTaO_3_ heterointerfaces is comparable to the bandwidth and the accompanying strong on-site Coulomb repulsion^25–27^, the combination of strong spin-orbit coupling and the electron-electron interaction is theoretically expected to result in an unconventional superconductivity, including a mix of spin-singlet and spin-triplet components^28^ as a manifestation of rotational symmetry breaking. Experimentally, an indication of strong in-plane anisotropic electrical resistance in the normal state has been reported at the ferromagnetic heterointerface of p-EuO/KTaO_3_, implying a possible existence of “stripe”-like superconducting phase^15^. This anisotropy, however, is alternatively attributed to be an extrinsic property of the ferromagnetic p-EuO in theory^29^, leading to that a consensus on the rotational symmetry breaking of superconductivity in KTaO_3_ heterointerface superconductors remains elusive.

Here, we carry out an experimental study on nonmagnetic a-YAlO_3_ thin films with a wide energy gap of 7.9 eV grown on the polar KTaO_3_(111) single-crystal substrates. This energy gap is significantly larger than that of a-LaAlO_3_ (5.6 eV)^30^, enabling strong confinement potential to restrict the interfacial conducting electrons to a thinner interfacial layer, thus prompting an intriguing quantum behaviors at their interface^31^. Electrical transport measurements on the as-grown films reveal two-dimensional superconductivity with a *T*_*c*_ of 1.86 K, and a superconducting layer thickness of 4.5 nm. By tuning the in-plane azimuthal angle *φ*-dependent magnetic field, both the magnetoresistance and superconducting critical field display pronounced twofold symmetric oscillations deep inside the superconducting state, whereas they vanish in the normal state. These results unambiguously demonstrate that the anisotropy with in-plane rotational symmetry breaking is an intrinsic property of the superconducting phase in a-YAlO_3_/KTaO_3_. Through group theory study, we thus classify the inversion symmetry breaking KTaO_3_ heterointerface superconductors as a mixed-parity unconventional superconductivity with an admixture of *s*-wave and *p*-wave pairing components, a candidate platform for realizing Majorana modes^32^.

## Results

**Fig. 1:**
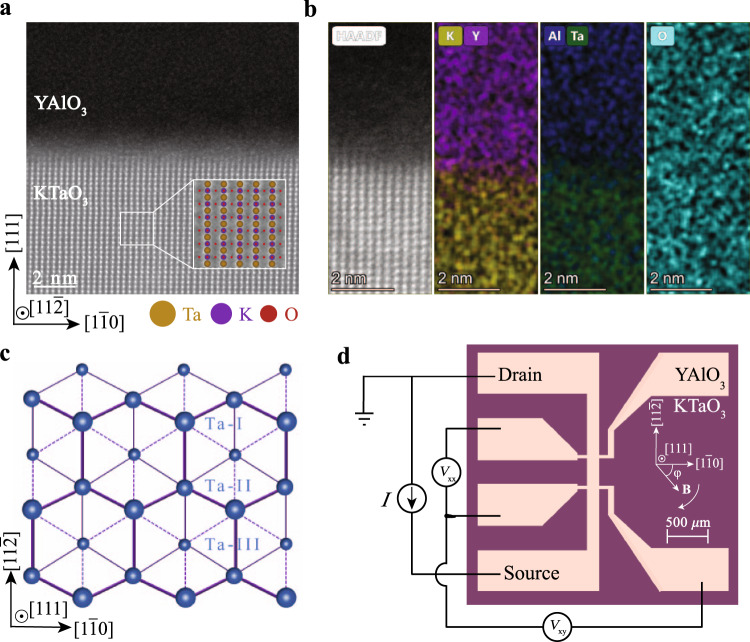
Structural and composition characterizations of a-YAlO_3_/KTaO_3_(111). **a** HAADF-STEM image of a-YAlO_3_/KTaO_3_ viewed along the $[11\bar{2}]$ zone axis. The inset shows the enlarged HR-STEM image of KTaO_3_ overlapped with atomic configuration. **b** HR-STEM image and the corresponding EDX elemental mapping of interface. **c** Distribution of Ta^5+^ ions along the [111] crystal axis of KTaO_3_(111) surface. Ta^5+^ ions are shown with progressively smaller sizes in the three adjacent (111) planes, which are labeled as Ta-I, Ta-II, and Ta-III, respectively. **d** Hall bar configuration on a-YAlO_3_/KTaO_3_(111) heterostructure. Here, *φ* is defined as the in-plane azimuthal angle between the applied magnetic field **B** and the $[1\bar{1}0]$-axis of the lattice, shown in the inset of **d**.

The a-YAlO_3_/KTaO_3_ heterostructures are prepared by depositing a-YAlO_3_ films on (111)-oriented KTaO_3_ single-crystal substrates using pulsed laser deposition. Atomic force microscopy characterizations show that the surface of KTaO_3_ substrates and a-YAlO_3_ films are atomically flat (see Supplementary Fig. 1). X-ray diffraction confirms the absence of epitaxial peaks of YAlO_3_ (see Supplementary Fig. 2 and Supplementary Fig. 3), thus suggesting that the YAlO_3_ film is not in a well-defined crystalline phase. The microstructure of the interface is further examined by aberration-corrected scanning transmission electron microscopy (STEM). From the high angle annular dark field (HAADF)-STEM image shown in Fig. 1a, it can be seen that the homogeneous and amorphous phase YAlO_3_ thin film is grown on the KTaO_3_(111) substrate (also see Supplementary Fig. 4). Looking at the sample from a larger field of view, the thickness of the a-YAlO_3_ film is found to be about 60 nm. High-resolution (HR)-STEM imaging shown in Fig. 1a and Supplementary Fig. 4, and energy dispersive X-ray spectroscopy (EDX) elemental mapping shown in Fig. 1b indicate that the abrupt and smooth interface between KTaO_3_ single-crystal substrate and a-YAlO_3_ film is resolved structurally and chemically. These results are consistent with previous studies on a-LaAlO_3_/KTaO_3_(111)^15,16^, a-LaAlO_3_/KTaO_3_(110)^23^, a-LaAlO_3_/KTaO_3_(001)^33^, and a-AlO_*x*_/KTaO_3_(111)^34^.

**Fig. 2:**
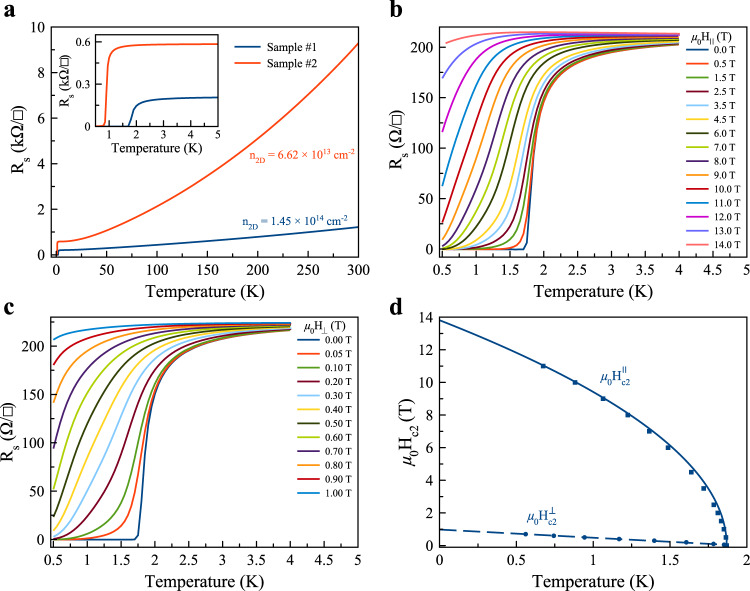
Superconducting properties of a-YAlO_3_/KTaO_3_(111). **a** Electrical resistance (R_s_) as a function of temperature at zero magnetic field for two representative a-YAlO_3_/KTaO_3_ heterostructures (Samples #1 and #2). Low temperature dependence of R_s_ is illustrated in the inset of **a**. Magnetoresistance for fields **b** parallel and **c** perpendicular to the plane surface of Sample #1. **d** Temperature dependence of the upper critical field *μ*_0_H_*c*2_ (*μ*_0_H${}_{c2}^{\parallel }$ for the in-plane field along the $[11\bar{2}]$-axis shown in Fig. 1d and *μ*_0_H${}_{c2}^{\perp }$ for the out-of-plane field).

Figure 2a shows the temperature-dependent sheet resistance R_s_ on two representative as-grown a-YAlO_3_ thin films (Samples #1 and #2 with growth temperatures of 780 and 650 °C, respectively) with the Hall bar configuration, schematically illustrated in Fig. 1d. A typical metallic behavior is visible in a wide temperature range, indicating that a two-dimensional electron gas is formed at their interface induced by a candidate mechanism of the formation of oxygen vacancies at the surface of KTaO_3_^23,35^, similar to that in the sister a-LaAlO_3_/SrTiO_3_^36^. The transverse Hall resistance R_xy_ is obtained from Hall measurements at 5 K, and reveals that the charge carriers in the a-YAlO_3_/KTaO_3_ are electrons. The estimated carrier density is about 1.45 × 10^14^ and 6.62 × 10^13^ cm^−2^ for Samples #1 and #2, respectively. The electron mobility for Samples #1 and #2 is thus 193.6 and 159.7 cm^2^V^−1^s^−1^. These results are highly universal and reproducible (see Supplementary Fig. 5, Supplementary Note 1, and Supplementary Table 1) and reasonably consistent with previous electrical transport studies on the KTaO_3_ heterointerfaces^15,23^. Remarkably, as the temperature is further decreased, the resistance R_s_ undergoes a narrow and sharp transition with a transition width of less than 0.5 K to a zero-resistance state, signaling the appearance of superconductivity at the heterointerface of a-YAlO_3_/KTaO_3_. The critical temperature is determined to be *T*_*c*_ = 1.86 and 0.92 K for Samples #1 and #2, respectively, as defined by where the resistance is at the midpoint of the normal electrical resistance at 5 K, i.e. R_s_(*T*_*c*_) = 0.5 × R_s_(5 K).

To further characterize the superconducting behaviors in a-YAlO_3_/KTaO_3_, we measure the magnetoresistance R_s_(*μ*_0_H) (here, *μ*_0_ is the vacuum permeability) at various temperatures between 0.5 and 5 K with fields parallel (*μ*_0_H_∥_) and perpendicular (*μ*_0_H_⊥_) to the plane surface of Sample #1, as shown in Fig. 2b, c, respectively. The fundamental superconducting behavior is clearly perceived. Indeed, the magnetoresistance R_s_(*μ*_0_H) varies differently with *μ*_0_H_∥_ and *μ*_0_H_⊥_, and both the upper critical fields *μ*_0_H${}_{c2}^{\parallel }$ and *μ*_0_H${}_{c2}^{\perp }$ parallelly shift to a lower value with the increase of the temperature, where *μ*_0_H_*c*2_ are evaluated at the midpoints of the normal state resistance at 5 K. These results provide an indication of a two-dimensional superconducting feature in a-YAlO_3_/KTaO_3_. The temperature-dependent upper critical fields *μ*_0_H_*c*2_ are summarized in Fig. 2d and are well fitted by the phenomenological two-dimensional Ginzburg-Landau (G-L) model^37^: *μ*_0_H${}_{c2}^{\perp }(T)=\frac{{{{\Phi }}}_{0}}{2\pi {\xi }_{{{{{{{{\rm{GL}}}}}}}}}^{2}}(1-\frac{T}{{T}_{c}})$ and *μ*_0_H${}_{c2}^{\parallel }(T)=\frac{{{{\Phi }}}_{0}\sqrt{12}}{2\pi {\xi }_{{{{{{{{\rm{GL}}}}}}}}}{d}_{{{{{{{{\rm{SC}}}}}}}}}}\sqrt{1-\frac{T}{{T}_{c}}}$, where Φ_0_, *ξ*_GL_, and *d*_SC_ denote a flux quantum, the in-plane superconducting coherence length at *T* = 0 K, and the effective thickness of superconductivity, respectively. Using the extrapolated *μ*_0_H${}_{c2}^{\perp }(0)$ = 0.98 T and *μ*_0_H${}_{c2}^{\parallel }(0)$ = 13.81 T, we find *ξ*_GL_ = 18.4 nm and *d*_SC_ = 4.5 nm, where *ξ*_GL_ is significantly larger than *d*_SC_, suggesting a two-dimensional nature of superconductivity. Additionally, the in-plane *μ*_0_H${}_{c2}^{\parallel }(0)$ is substantially larger than the Pauli-paramagnetic pair-breaking field B_P_ ≈ 3.46 T based on the BCS theory in the weak-coupling limit^38,39^. High values of *μ*_0_H${}_{c2}^{\parallel }(0)$ excessing B_P_ could be realized in the presence of strong spin-orbit coupling owing to the elastic scattering, which results in the suppression of spin paramagnetism effects. The violation of this paramagnetic limit is a common phenomenon in heterointerface superconductors^15,40^, especially when the superconducting layer thickness is in the range *d*_SC_ < 20 nm. However, the underlying mechanism for realizing *μ*_0_H${}_{c2}^{\parallel }(0)$ value in excess of B_P_ remains an open question^15^. Furthermore, the thickness of the superconducting layer in a-YAlO_3_/KTaO_3_(111) is approximately estimated as thin as *d*_SC_ = 4.5 nm based on the framework of the phenomenological two-dimensional G-L model^37^. This result could be intuitively expected, since the strong confinement potential induced by YAlO_3_ significantly restricts the superconducting electrons to a thinner superconducting layer^31^. On the other hand, the out-of-plane polar angle *θ*-dependent upper critical field H${}_{c2}^{\theta }$ at 1.5 K quantitatively verifies the behavior expected from a two-dimensional structure in a-YAlO_3_/KTaO_3_, as shown in Supplementary Fig. 6. The *θ*-dependent *μ*_0_H${}_{c2}^{\theta }$ are quantitatively fitted by the two-dimensional Tinkham formula and the three-dimensional anisotropic G-L model, given by $\frac{{{{{{{{{\rm{H}}}}}}}}}_{c2}^{\theta }|\cos \theta|}{{{{{{{{{\rm{H}}}}}}}}}_{c2}^{\perp }}+{(\frac{{{{{{{{{\rm{H}}}}}}}}}_{c2}^{\theta }\sin \theta }{{{{{{{{{\rm{H}}}}}}}}}_{c2}^{\parallel }})}^{2}=1$ and ${(\frac{{{{{{{{{\rm{H}}}}}}}}}_{c2}^{\theta }\cos \theta }{{{{{{{{{\rm{H}}}}}}}}}_{c2}^{\perp }})}^{2}+{(\frac{{{{{{{{{\rm{H}}}}}}}}}_{c2}^{\theta }\sin \theta }{{{{{{{{{\rm{H}}}}}}}}}_{c2}^{\parallel }})}^{2}=1$, respectively^41,42^. A cusp-like peak is clearly observed at *θ* = 90° (see Supplementary Fig. 6), which is well described by the two-dimensional Tinkham model, as frequently observed in heterointerface superconductivity^42,43^ and layered transition metal dichalcogenides^44,45^.

**Fig. 3:**
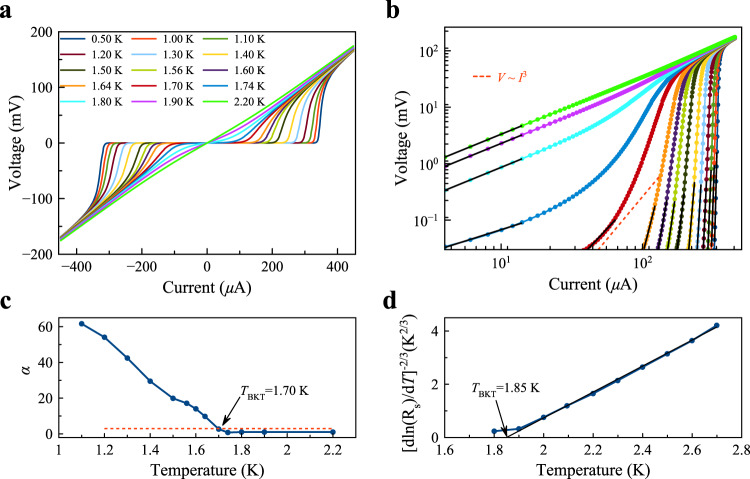
Two-dimensional superconducting behavior of a-YAlO_3_/KTaO_3_(111). **a** Temperature-dependent *I*-*V* measurements (Sample #1). **b** Corresponding logarithmic scale representation of **a**. The long red dashed line denotes the *V* ~ *I*^3^ dependence. **c** Temperature dependence of the power-law exponent *α*, as deduced from the fits shown in **b**. **d** R_s_(*T*) dependence of the same sample, plotted on a ${[{{{\rm{d}}}}{{{\rm{ln}}}}({{{\rm{R}}}}_{{{\rm{s}}}})/{{{\rm{d}}}}T]}^{-2/3}$ scale.

Since the superconductivity in a-YAlO_3_/KTaO_3_ is two-dimensional, the Berezinskii-Kosterlitz-Thouless (BKT) transition describes superconducting phase coherence^46,47^. Here, the BKT transition temperature defines the vortex unbinding transition, and can be determined using current-voltage (*I*-*V*) measurements as a function of temperature *T*, as shown in Fig. 3a. Below *T*_*c*_, we find a critical current *I*_*c*_, whose value decreases with increase in temperature. The maximal value of *I*_*c*_ is ~ 330 *μ*A at 0.5 K, which is substantially larger than that previously observed in the KTaO_3_ heterointerfaces^15,23^. Such a high critical current value probably originates from the high charge carrier concentration (about 1.45 × 10^14^ cm^−2^, Sample #1 in Fig. 2a) confined in a thinner superconducting layer of a-YAlO_3_/KTaO_3_, promising for large-scale applications in superconductor-based devices. In Fig. 3b, we also plot the characteristics *I*-*V* on a log-log scale, and observe that the slope of the *I*-*V* curve smoothly evolves from the normal ohmic state, *V* ∝ *I*, to a steeper power law resulting from the current exciting free-moving vortices, *V* ∝ *I*^*α*(*T*)^, with *α*(*T*_BKT_) = 3. In Fig. 3c, a value *T*_BKT_ = 1.7 K is interpolated, which is consistent with *T*_*c*_ as defined in Fig. 2a. In addition, close to *T*_BKT_, an R${}_{{{{{{{{\rm{s}}}}}}}}}={R}_{0}\exp [-b{(T/{T}_{{{{{{{{\rm{BKT}}}}}}}}}-1)}^{-1/2}]$ dependence, where *R*_0_ and *b* are material parameters, is expected^48^. As shown in Fig. 3d, the measured R_s_(*T*) is also consistent with this behavior and yields *T*_BKT_ = 1.85 K, in good agreement with the analysis of the *α* exponent shown in Fig. 3c.

**Fig. 4:**
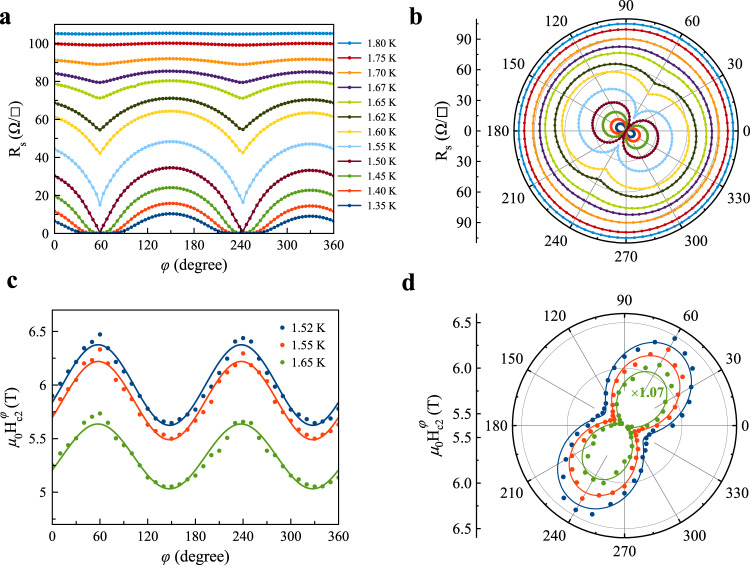
In-plane twofold symmetric oscillations in a-YAlO_3_/KTaO_3_(111). **a** In-plane angular-dependent magnetoresistance R_s_ at various temperatures for an applied field of 1 T. **b** Polar plots of the data in **a**. **c** In-plane angular-dependent *μ*_0_H${}_{c2}^{\varphi }$ at various temperatures. **d** Polar plots of the data in **c**. The solid lines in **c** and **d** are the theoretical fits of the H${}_{c2}^{\varphi }$ using the gap function of ∣Δ_gap_∣^2^ with an admixture of *s*-wave and *p*-wave pairings, ${{{\Delta }}}_{{{{{{{{\rm{gap}}}}}}}}}({{{{{{{\bf{k}}}}}}}})=i[{{{\Delta }}}_{s}{\hat{\sigma }}_{0}+{{{\Delta }}}_{p}\sin ({k}_{x}){\hat{\sigma }}_{3}]{\hat{\sigma }}_{2}$. Here, Δ_*s*_ and Δ_*p*_ are the pairing amplitudes of *s*-wave and *p*-wave, respectively, $\hat{\sigma }$ is the vector of Pauli matrices, and **k** is the momentum.

Next, we turn to discuss the in-plane anisotropy of superconductivity in a-YAlO_3_/KTaO_3_ using an in-plane azimuthal angle *φ*-dependent magnetoresistance, where *φ* is defined as the azimuthal angle between the magnetic field and the $[1\bar{1}0]$-axis of the lattice, as indicated in Fig. 1d. Care has been taken to rule out the inevitable misalignment effects of an accidental out-of-plane component of the field, when the vector magnet is utilized. In the normal state (*T* = 1.8 K in Fig. 4a) of Sample #4 (Supplementary Fig. 7), the magnetoresistance R_s_ is found to be essentially independent of *φ*, displaying an isotropic behavior. Whereas in the superconducting state (*T* = 1.5 K in Fig. 4a), we observe a pronounced twofold symmetric oscillations of the R_s_ (see Fig. 4b), which is consistent across multiple samples including the heterointerfaces of a-YAlO_3_/KTaO_3_(111) and sister a-LaAlO_3_/KTaO_3_(111) (see Supplementary Fig. 8 − Supplementary Fig. 10 and Supplementary Note 2). In this case, the anisotropic R_s_ attains the maximum value when the magnetic field is directed along the special $[1\bar{2}1]$-axis (*φ* = − 30° or 150°) that is in the direction of one of the principal axes of KTaO_3_(111) shown in Fig. 1c, and becomes minimum when the position with respect to that of maximum is shifted by 90° (*φ* = 60° or 240°). This finding implies that an extrinsic error from the experimental setup is unlikely to the source of the observed twofold anisotropy of magnetoresistance in the superconducting state at the heterointerface of a-YAlO_3_/KTaO_3_. In particular, the significantly large anisotropic ratio of R_s_(*φ* = 60°)/R_s_(*φ* = 150°) = 0.03 at 1.5 K corresponds to a putative misalignment angle estimated up to 88.03° ($\cos$ 88.03° = 0.03) between the field and the basal plane^49^, which is impossible for such a large angle misalignment in the vector magnet, we could exclude the possible contribution from an accidental misalignment of the field with the film plane. Since the magnetoresistance minima approach zero in R_s_(*φ*) curve measured at 1.5 K (see Fig. 4a), and considering the fact that the existence of conspicuous twofold symmetry in magnetoresistance manifests deep inside the superconducting region, which vanishes in the normal state (see Fig. 4a and Supplementary Fig. 11), we could further rule out the possibilities of extrinsic contributions, such as the magnetic field induced Lorentz force effect^50^ and the Fermi surface inherent to the KTaO_3_ with respect to the underlying threefold lattice symmetry^26^ (Fig. 1c and Supplementary Fig. 3), and thus demonstrate that this anisotropy with rotational symmetry breaking is an intrinsic property of the superconducting phase in a-YAlO_3_/KTaO_3_.

To further reveal the twofold symmetric superconductivity in a-YAlO_3_/KTaO_3_ in terms of the superconducting gap structure, we extract the upper critical field *μ*_0_H_*c*2_ from the *φ*-dependent magnetoresistance R_s_ in the superconducting region determined by the criterion of 90% sheet resistance dropped from normal state, as shown in Fig. 4c. Here, it should be pointed out that although the values of H_*c*2_ are changed by different criteria, the symmetry of H_*c*2_ itself remains qualitatively (see Supplementary Fig. 12). In addition, it should be noted that the data shown here have been taken by averaging the raw data with positive and negative magnetic fields to avoid the possible asymmetric problem. Remarkably, the in-plane *φ*-dependent *μ*_0_H${}_{c2}^{\varphi }$ also displays twofold symmetric oscillations (see Fig. 4d), providing additional strong evidence for the twofold rotational symmetry of the superconductivity in a-YAlO_3_/KTaO_3_. Furthermore, the oscillation of *μ*_0_H${}_{c2}^{\varphi }$ has a *π* phase shift compared with that of the R_s_ (see Fig. 4b) such that for the *φ* value where superconductivity is hardest to suppress, *μ*_0_H${}_{c2}^{\varphi }$ is the largest and R_s_ is the lowest (Fig. 4b, d), as expected from our intuitions^50–52^. Since *μ*_0_H${}_{c2}^{\varphi }$ achieves its maximum for the field applied perpendicular to the main crystallographic axis, and minimum for the direction along the main crystallographic axis (see Figs. 1d and 4d), the superconducting gap leads to a maximum (or minimum) direction perpendicular (or parallel) to the main crystallographic axis, manifesting a rotational symmetry breaking state of superconducting a-YAlO_3_/KTaO_3_ with the direction of the minimum gap spontaneously pinned to the main crystallographic axis.

## Discussion

Having experimentally established the intrinsic twofold anisotropy of the superconducting state of a-YAlO_3_/KTaO_3_, we now proceed to elaborate about its origin using the underlying symmetries of the crystal structure without requiring the details of the pairing mechanisms based on the group theoretical formulation of the Ginzburg-Landau theory^53^(also see Supplementary Note 2 and Supplementary Note 3 in details). This allows us to deduce fundamental information about the superconducting ground state in the a-YAlO_3_/KTaO_3_ heterointerface superconductors. From the viewpoint of group symmetry, if a superconductor possesses an inversion symmetry, the Pauli principle requires a totally antisymmetric Cooper pair wavefunction, which imposes the condition that the superconducting states should be either spin-singlet or spin-triplet, whereas mixed-parity states are forbidden^53^. In the a-YAlO_3_/KTaO_3_ the lack of inversion symmetry, however, tends to mix spin-singlet and spin-triplet driven by strong spin-orbit coupling^54^. Indeed, the conducting electrons with strong spin-orbit coupling originating from the heavy Ta 5*d* orbitals has been elucidated at the KTaO_3_ heterointerfaces^26,33,55–60^ (also see Supplementary Fig. 13). Due to the absence of a mirror plane parallel to the interface of a-YAlO_3_/KTaO_3_, the point group of a-YAlO_3_/KTaO_3_ is *C*_3*v*_, which does not contain the symmetry element of an inversion. This situation is analogue to non-centrosymmetric superconductors^54,61^. Upon inspecting the character table of *C*_3*v*_ point group tabulated in Supplementary Table 2, we notice that the mixed-parity superconducting state only belongs to the *A*_1_+*E*-representation with the possible basis function of *s*+*p*. Notably, the two-dimensional irreducible representation of *E* could spontaneously break the threefold rotational symmetry of the crystal (see Fig. 1c and Supplementary Fig. 3), leading to a subsidiary uniaxial anisotropy or nematic superconductivity^62,63^, such as a uniaxial *p*_*x*_-wave or *p*_*y*_-wave pairing. Since the upper critical field is proportional to the square of the superconducting gap amplitude based on the Ginzburg-Landau theory and the Pippard definition of the coherence length^49^, *μ*_0_H${}_{c2}^{\varphi }\propto|{{{\Delta }}}_{{{{{{{{\rm{gap}}}}}}}}}(\varphi ){|}^{2}$, only the *s*+*p*_*x*_-wave pairing with the gap function of ${{{\Delta }}}_{{{{{{{{\rm{gap}}}}}}}}}({{{{{{{\bf{k}}}}}}}})=i[{{{\Delta }}}_{s}{\hat{\sigma }}_{0}+{{{\Delta }}}_{p}\sin ({k}_{x}){\hat{\sigma }}_{3}]{\hat{\sigma }}_{2}$ (here, Δ_*s*_ and Δ_*p*_ are the pairing amplitudes of *s*-wave and *p*-wave, respectively, $\hat{\sigma }$ is the vector of Pauli matrices, and **k** is the momentum)^61,64^, could give rise to an overall twofold anisotropic gap and well reproduce the exact topology of the anisotropic H${}_{c2}^{\varphi }$ shown in Fig. 4d. Therefore, the mix of *s*-wave and *p*-wave pairings driven by strong spin-orbit coupling is suggested to the source of the experimentally observed twofold anisotropic superconductivity at the KTaO_3_ heterointerfaces, which has long been a topic of interest sought in condensed matter physics. Further experiments, including probes of the superconducting gap by tunneling spectroscopy and/or Josephson junction experiments, will also be helpful for clarifying the underlying mixed-parity pairing nature of the twofold symmetric superconductivity that we observe.

In summary, we have experimentally observed spontaneous rotational symmetry breaking from threefold to twofold in the superconducting state of KTaO_3_(111) heterointerfaces with respect to an application of in-plane magnetic field. This in-plane anisotropic superconductivity is theoretically attributed to the intrinsic nature of mixed-parity unconventional superconductivity with an admixture of *s*-wave and *p*-wave pairing components, bringing with it fresh new insights into the study of emergent fascinating and non-trivial superconducting properties at the heterointerfaces with inversion symmetry breaking.

## Methods

### Thin film growth and structural characterizations

a-YAlO_3_ thin films are grown on KTaO_3_(111) single-crystal substrates (5 × 5 × 0.5 mm^3^) by pulsed laser deposition in an ultrahigh vacuum chamber (base pressure of 10^−9^ Torr). Prior to growth, the KTaO_3_ substrates are annealed at 600 °C for 30 mins in ultrahigh-vacuum to obtain a smooth surface (Supplementary Fig. 1). During deposition, a single crystal YAlO_3_ target (Kurt J. Lesker Company) is used to grow the a-YAlO_3_ films with a KrF excimer laser (Coherent 102, wavelength: *λ* = 248 nm). A pulse energy density of 1.5 J/cm^2^ and a repetition rate of 2 Hz are used. The a-YAlO_3_ films are deposited at temperatures ranging from 600 to 900 °C in a vacuum chamber to promote growth of the superconducting phase. All the samples are cooled to room temperature at a constant rate of 20 °C/min in vacuum after deposition. The quality of a-YAlO_3_ films under ambient conditions is examined by atomic force microscopy (AFM, Asylum Research MFP-3D Classic) and by four-circle X-ray diffraction (XRD, Bruker D8 Discover, Cu K*α* radiation, *λ* = 1.5406 Å) operated in HR mode using a three-bounce symmetric Ge (022) crystal monochromator.

### STEM measurements

Cross-sectional specimens for electron microscopy are prepared with Focused Ion Beam (FIB) (Helios-G4-CX, Thermo Fisher Scientific) using lift-out method. The HR-STEM images are performed on a double aberration corrected field-emission STEM (Themis Z, Thermo Fisher Scientific) operated at 300 kV. For HAADF-STEM imaging, the semi-convergent angle of the probe forming lens is set to 21.4 mrad. The geometric aberrations within the probe forming lens aperture have been effectively tuned to zero using probe corrector (SCORR, CEOS GmbH). The semi-collection angle of the HAADF detector is 76–200 mrad. Furthermore, the chemical composition of the interface is qualitatively analyzed using EDX in STEM spectrum imaging mode. The EDX are collected using 4 silicon drift detector (SDD) system (Super X detector, Thermo Fisher Scientific). The beam current for STEM-EDX analysis is about 200 pA.

### Electrical transport measurements

The electrical transport measurements are performed using a commercial cryostat with temperature ranging from 1.5 to 300 K (Oxford Instruments TeslatronPT cryostat system), physical properties measurement system with temperature ranging from 0.5 to 300 K (PPMS, Quantum Design), and 10 mK dilution refrigerator with vector magnet (Oxford Instruments Triton 200). The Hall bar structure (Fig. 1d) is fabricated by ion-beam etching to systemically measure the electrical transport properties. The vector magnet is utilized to reveal the in-plane anisotropy of magnetoresistance in the superconducting state shown in Fig. 4a, and the samples are mounted on a mechanical rotator in a ^4^He cryostat to clarify the anisotropy of H_*c*2_ shown in Fig. 4c. The misalignment of the field with the film plane is estimated to be less than 2° and 7° for vector magnet and mechanical rotator, respectively, as our experimental errors.

## Supplementary information

Supplementary Information

## Data Availability

The relevant data supporting our key findings are available within the article and the Supplementary Information file. All raw data generated during our current study are available from the corresponding authors upon reasonable request.
