# Supplementary material for: Spontaneous rotational symmetry breaking in KTaO3 heterointerface superconductors
Source: Nat Commun. 2023 May 26;14:3046. doi: 10.1038/s41467-023-38759-0 (PMC10220087; doi:10.1038/s41467-023-38759-0)
Supplement: Supplementary file 1 — Supplementary Information [file 41467_2023_38759_MOESM1_ESM.pdf]

Supplementary Information for:

Spontaneous rotational symmetry breaking in $KTaO_3$ heterointerface superconductors

Guanqun Zhang,[1] Lijie Wang,[1] Jinghui Wang,[2] Guoan Li,[3] Guangyi Huang,[1] Guang Yang,[3] Huanyi Xue,[1] Zhongfeng Ning,[1] Yueshen Wu,[2] Jin-Peng Xu,[3] Yanru Song,[4,*] Zhenghua An,[1,5] Changlin Zheng,[1] Jie Shen,[3,6*] Jun Li,[2,*] Yan Chen,[1] and Wei Li[1,*]

[1]*State Key Laboratory of Surface Physics and Department of Physics, Fudan University, Shanghai 200433, China.*

[2]*ShanghaiTech Laboratory for Topological Physics & School of Physical Science and Technology, ShanghaiTech University, Shanghai 201210, China.*

[3]*Beijing National Laboratory for Condensed Matter Physics and Institute of Physics, Chinese Academy of Sciences, Beijing 100190, China.*

[4]*ShanghaiTech Quantum Device Lab, ShanghaiTech University, Shanghai 201210, China.*

[5]*Institute for Nanoelectronic Devices and Quantum Computing, Fudan University, Shanghai 200433, China.*

[6]*Songshan Lake Materials Laboratory, Dongguan 523808, China.*

G.Z., L.W., J.W., and G.L. contributed equally to this work.

*Correspondence and requests for materials should be addressed to Yanru Song (Email: songyr@shanghaitech.edu.cn), Jie Shen (Email: shenjie@iphy.ac.cn), Jun Li (Email: lijun3@shanghaitech.edu.cn), or Wei Li (Email: w_li@fudan.edu.cn).

This file includes:

Supplementary Information Text: Supplementary Note 1 – Supplementary Note 3
Supplementary Fig. 1 – Supplementary Fig. 13
Supplementary Table 1 – Supplementary Table 2
SI References

Supplementary Information Text

Supplementary Note 1. Additional experimental data for the intrinsic interfacial superconductivity in the $KTaO_3$(111) heterostructures.

To further support the intrinsic interfacial superconductivity in the $KTaO_3$(111) heterostructures, we carry out the measurements of the thickness-dependent thin films of the amorphous (a)-$YAlO_3$ with the thicknesses of 7 nm, 15 nm, and 32 nm grown on the $KTaO_3$(111) substrates, as shown in Supplementary Fig. 5. Interestingly, the low temperature-dependent electrical transport measurements reveal that these three samples all exhibit a narrow and sharp transition to a zero-resistance state, signaling the appearance of superconductivity at the $KTaO_3$(111) heterointerfaces, even in a thinner film of the a-$YAlO_3$ with the thickness of 7 nm, indicative of the intrinsic interfacial superconducting nature of the a-$YAlO_3$/$KTaO_3$(111).

Furthermore, we have also grown the sister thin films of the a-$LaAlO_3$ on the $KTaO_3$(111) substrates using the same growth conditions to that of a-$YAlO_3$ films described in the Methods, and found the conspicuous signal of the appearance of superconductivity in the a-$LaAlO_3$/$KTaO_3$(111) heterostructure (Sample #6), as shown in Supplementary Fig. 9a, similar to that of a-$YAlO_3$/$KTaO_3$(111) shown in Supplementary Fig. 5. Therefore, these independent and complementary electrical transport results on multiple samples, including the a-$LaAlO_3$/$KTaO_3$(111) and a-$YAlO_3$/$KTaO_3$(111) heterostructures, provide the strong compelling evidence for the intrinsic interfacial superconductivity in the $KTaO_3$ heterostructures.

Supplementary Note 2. Additional experimental data for the intrinsic twofold symmetric magnetoresistance observed in the superconducting state of the $KTaO_3$(111) heterointerfaces.

To further shed light on the intrinsic twofold rotational symmetric magnetoresistance observed in the superconducting state of the $KTaO_3$(111) heterointerfaces (Fig. 4), we have fabricated two perpendicular Hall bar devices along the $[11\bar{2}]$ and $[1\bar{1}0]$ orientations in the same sample of the a-$YAlO_3$/$KTaO_3$(111) heterostructure (Sample #7), as shown in Supplementary Fig. 10. Interestingly, the measured charge carrier density is about 7.1×10^{13} cm^{-2} for both orientations, as shown in Supplementary Fig. 10d, suggestive of the highly homogeneous sample of the a-$YAlO_3$/$KTaO_3$(111). Furthermore, the striking twofold anisotropic magnetoresistance in the superconducting state is clearly visible, whereas this anisotropy vanishes in the normal state, by using a dilution refrigerator with the vector magnet that could actively minimize the possible influence of field misalignment (see Supplementary Fig. 10e,f). Remarkably, the anisotropic R_s attains the maximum value when the magnetic field is directed along the special $[11\bar{2}]$ axis (φ =-30° or 150°) that is in the direction of one of the principal axes of the $KTaO_3$(111) heterostructures shown in Fig. 1c, and becomes minimum when the position with respect to that of maximum is shifted by 90° (φ = 60° or 240°), where the φ is defined as the in-plane azimuthal angle between the applied field and the $[1\bar{1}0]$-axis of the lattice shown in Supplementary Fig. 10a. These experimental results are consistent with the findings of the a-$YAlO_3$/$KTaO_3$(111) shown in Fig. 4 (Sample #4) and Supplementary Fig. 8 (Sample #5) and the sister a-$LaAlO_3$/$KTaO_3$(111) shown in Supplementary Fig. 9 (Sample #6), and can be regarded as the crucial characteristics that distinguish the intrinsic superconducting nature from a possible extrinsic effect. Therefore, these intriguing electrical transport results on the homogeneous thin film samples provide the strong additional

evidence for the intrinsic nature of the twofold symmetric superconductivity at the $KTaO_3(111)$ heterointerfaces.

Supplementary Note 3. Additional discussions on the underlying intrinsic nature of the twofold symmetric superconductivity observed at the $KTaO_3(111)$ heterointerfaces.

In the $KTaO_3(111)$ heterointerface superconductors, both the magnetoresistance R_s and superconducting critical field μ_0H_{c2} exhibit pronounced twofold symmetric oscillations deep inside the superconducting state, whereas the anisotropy vanishes in the normal state, by tuning the in-plane azimuthal angle φ-dependent magnetic fields, as shown in Fig. 4, demonstrating that this twofold rotational symmetry is an intrinsic property of the superconducting phase of the $KTaO_3(111)$ heterointerfaces (also see the detailed discussions in Supplementary Note 2). Theoretically, to the best of our knowledge, the nature of this intriguing twofold symmetric superconductivity could be understood through the following two possible mechanisms of (i) the Fulde-Ferrell-Larkin-Ovchnnikov (FFLO) state with an anisotropic finite momentum pairing, and (ii) the "stripe"-like phase or nematic superconductivity. Previously, the FFLO state has ever been theoretically proposed for the sister heterostructures[SI1], namely the crystalline (c)-$LaAlO_3/SrTiO_3(001)$, since the coexistence of ferromagnetism and superconductivity has been experimentally reported in the c-$LaAlO_3/SrTiO_3$[SI2-SI4]. This mechanism of FFLO state is, however, unlikely to the source of the present experimentally observed phenomenon in the $KTaO_3$ heterostructures. This is because of the following reasons:

(1) The FFLO state could be stabilized at the superconducting phase transition by the cooperative effects of the spin-orbit coupling and the applied in-plane field induced Zeeman splitting, since the twofold symmetric superconductivity at the $KTaO_3$ heterointerfaces is experimentally observed with an application of the in-plane field. This intriguing FFLO state will behave an anisotropic gap function with a periodic modulation of pairing order parameters in real space with wavelength in the order of coherence length[SI5]. Interestingly, if we further rotate the direction of the applied in-plane field on the basal plane of the $KTaO_3(111)$ heterointerfaces, this anisotropy of the superconducting pairing will exhibit sixfold rotational symmetry, preserving the underlying lattice symmetry of the basal plane of the $KTaO_3(111)$ heterointerfaces shown in Fig. 1c and Supplementary Fig. 3. However, in the present experimental data shown in Fig. 4, we find both the magnetoresistance R_s and the superconducting critical field μ_0H_{c2} in an in-plane field manifest striking twofold symmetric oscillations in the superconducting state that breaks the underlying rotational symmetry of the basal plane of the $KTaO_3(111)$ heterointerfaces. Therefore, this possibility of FFLO state could be excluded as the source of the experimentally observed twofold anisotropy in both the magnetoresistance R_s and the upper critical field μ_0H_{c2} in the superconducting state of the $KTaO_3$ heterointerfaces.

(2) The twofold symmetric superconductivity observed in the superconducting state of the $KTaO_3$ heterointerfaces could be explained as a "stripe"-like phase, namely nematic superconductivity, that breaks the underlying rotational symmetry of the lattice of the crystal. This nematic superconductivity is further explained theoretically as a consequence of the superconducting order parameter derived from the spin-orbit coupling and the multi-orbital effect[SI6,SI7]. Interestingly, the $KTaO_3$ heterointerfaces achieve both of these conditions, which are responsible for the emergence of the nematic superconductivity. By using the symmetry analysis based on the group theory (see the detailed discussions in the main text and Supplementary Table

2), we find the mixed-parity superconducting state with an admixture of *s*-wave and *p*-wave pairing components to be the ground state of the $KTaO_3$ heterointerface superconductors, consistent with the experimental results shown in Fig. 4. Therefore, we attribute this twofold rotational symmetric superconductivity to be the intrinsic nature of the mixed-parity superconductivity. Further experiments, including probes of the superconducting gap by tunneling spectroscopy and/or Josephson junction experiments, will also be helpful for elucidating the underlying mixed-parity pairing nature of the twofold symmetric superconductivity that we observe.

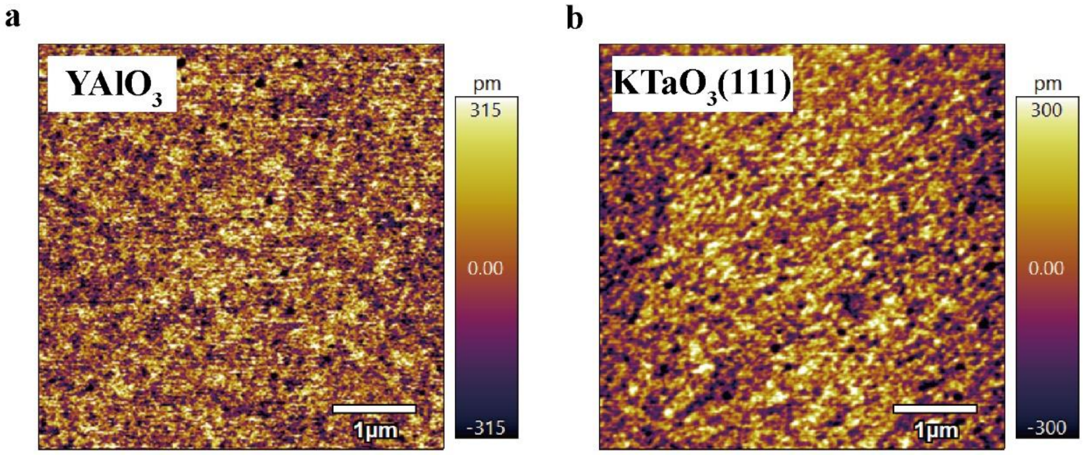

Supplementary Fig. 1| Large-area AFM images of a-$YAlO_3$ thin films and bare $KTaO_3$(111) single-crystal substrates. Large-area AFM images of **a** a-$YAlO_3$ thin film grown on the $KTaO_3$(111) single-crystal substrate and **b** bare $KTaO_3$(111) single-crystal substrate by using AFM (atomic force microscopy, Asylum Research MFP-3D Classic). These results indicate that the low lattice mismatch between a-$YAlO_3$ and $KTaO_3$ gives rise to the atomically flat surface of films, suggestive of homogeneous growth of a-$YAlO_3$ thin films on the $KTaO_3$ single-crystal substrates.

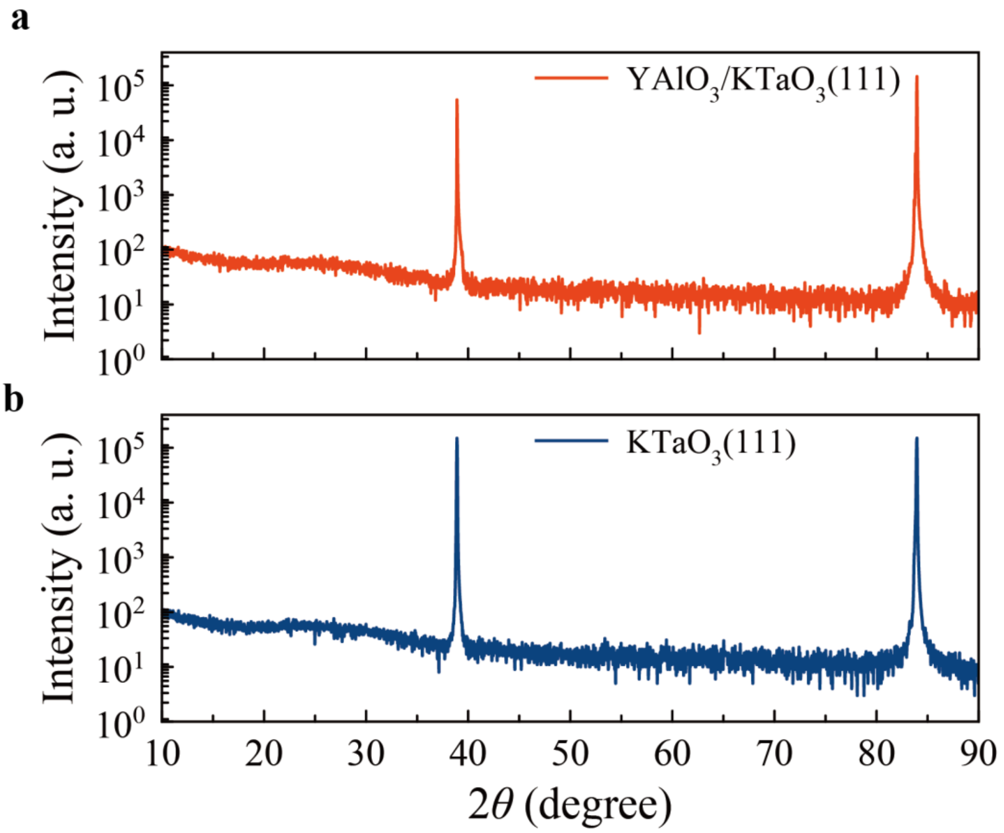

Supplementary Fig. 2| The θ-2θ scans of XRD for a-$YAlO_3$/$KTaO_3$(111) samples and bare $KTaO_3$(111) single-crystal substrates. The θ-2θ scans of **a** a-$YAlO_3$/$KTaO_3$(111) sample and **b** bare $KTaO_3$(111) single-crystal substrate using four-circle XRD (X-ray diffraction, Bruker D8 Discover, Cu Kα radiation, $\lambda = 1.5406$ Å) operated with high-resolution mode using a three-bounce symmetric Ge (022) crystal monochromator. Both peaks are assigned to the diffraction from the bare $KTaO_3$ single-crystal substrates, and no epitaxial peak from a-$YAlO_3$ film is detected, demonstrating that the amorphous phase of the $YAlO_3$ film is grown on the $KTaO_3$(111) single-crystal substrate.

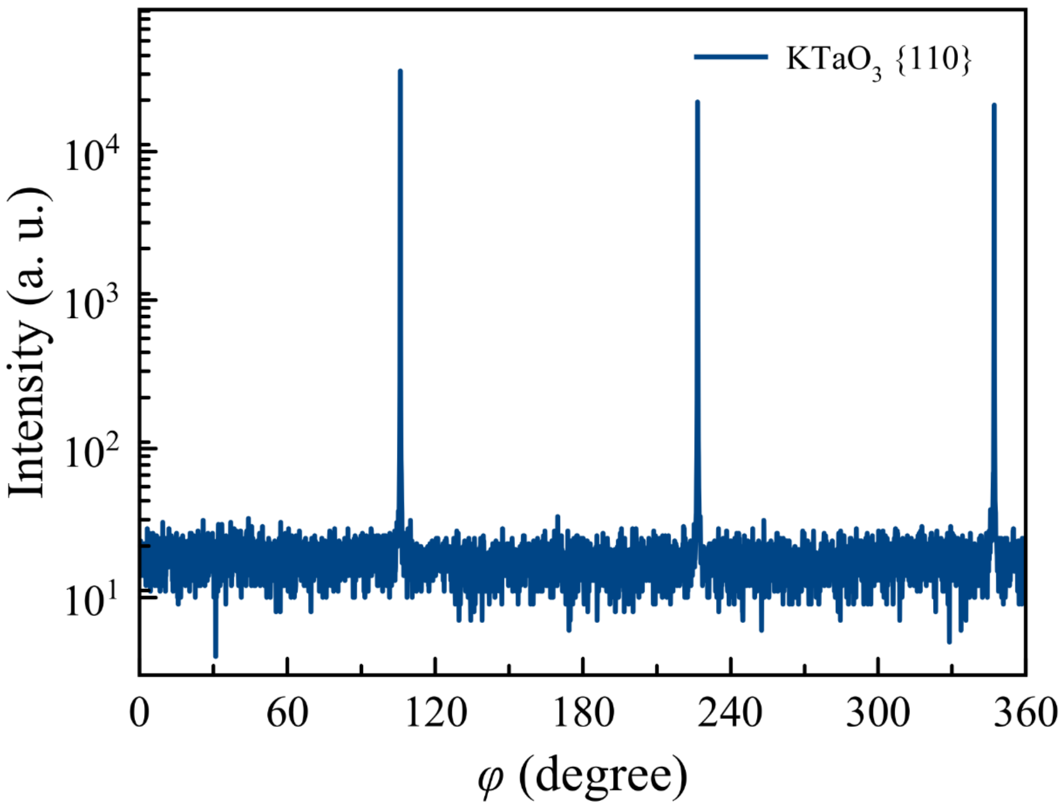

Supplementary Fig. 3| In-plane φ-scan of the {110} diffraction planes for the $KTaO_3$(111) heterointerfaces. The three peaks are uniformly distributed, displaying an in-plane threefold rotational symmetry of the lattice.

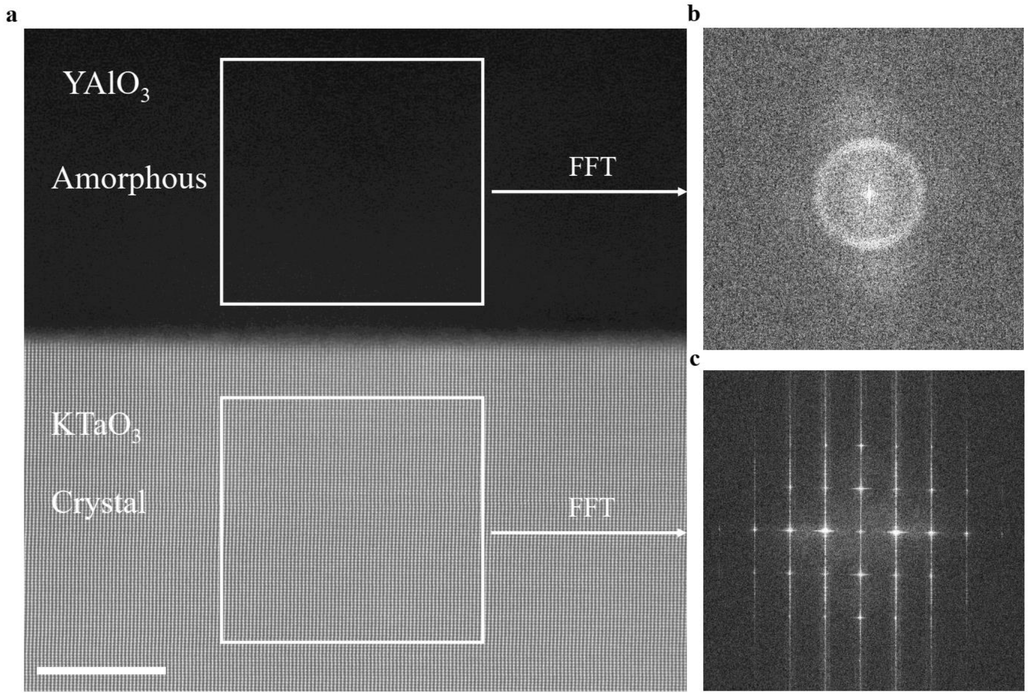

Supplementary Fig. 4| Large-scale HR-HAADF STEM image on a-$YAlO_3$/$KTaO_3$(111) heterostructures. **a** Large-scale high-resolution HAADF-STEM image taken of the a-$YAlO_3$/$KTaO_3$(111) heterostructure. Fast Fourier transform (FFT) images from the heterostructure are shown as insets, demonstrating that **b** the $YAlO_3$ film is amorphous, and **c** the $KTaO_3$(111) substrate near the interface is in a well-crystalline state.

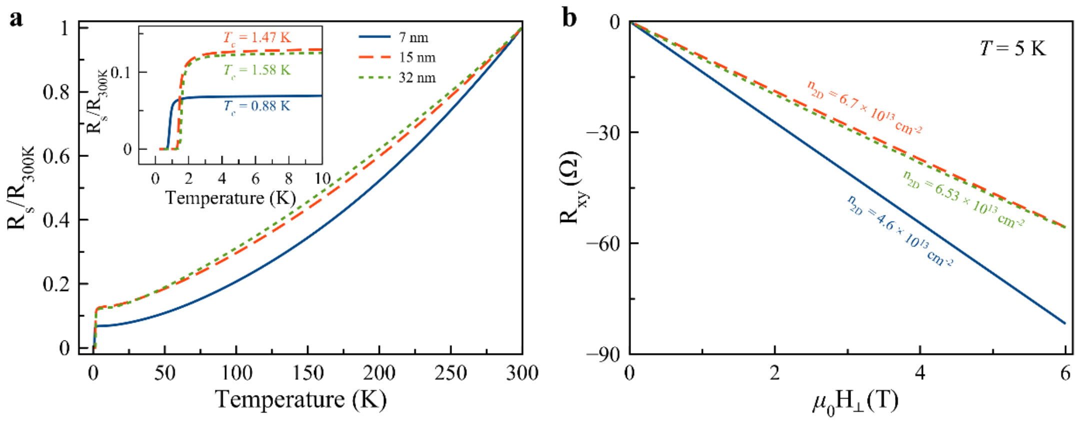

Supplementary Fig. 5| Thickness-dependent electrical properties for a-$YAlO_3$/$KTaO_3$(111) heterostructures. **a** Thickness-dependent rescaled electrical resistance R_s/R_{300K} for a-$YAlO_3$/$KTaO_3$(111) with growth temperature of 780 °C. R_{300K} denotes the electrical resistance R_s measured at a temperature of 300 K in the absence of field. Low temperature-dependent rescaled R_s/R_{300K} is illustrated in the inset of (a). The superconducting critical transition temperature T_c is defined by where the resistance is at the midpoint of the normal electrical resistance at 5 K. **b** Corresponding perpendicular field ($\mu_0 H_\perp$)-dependent transverse Hall resistance R_{xy} measured at the temperature of 5 K. Here, the thickness of the a-$YAlO_3$ films is measured by using an AFM (Asylum Research MFP-3D Classic).

Supplementary Table 1. Summary of sample properties.

No.	Samples	Growth T	n_{2D} (10^{13} cm^{-2})	T_c	Reference Figure
1	$YAlO_3/KTaO_3$, Sample #1	780 °C	14.5	1.86	Fig. 1a
2	$YAlO_3/KTaO_3$, Sample #2	650 °C	6.62	0.92	Fig. 1a
3	$YAlO_3/KTaO_3$, thickness of 7 nm	780 °C	4.6	0.88	Supplementary Fig. 5
4	$YAlO_3/KTaO_3$, thickness of 15 nm	780 °C	6.7	1.47	Supplementary Fig. 5
5	$YAlO_3/KTaO_3$, thickness of 32 nm	780 °C	6.53	1.58	Supplementary Fig. 5
6	$YAlO_3/KTaO_3$, Sample #3	780 °C	23.2	1.84	Supplementary Fig. 6
7	$YAlO_3/KTaO_3$, Sample #4	785 °C	31	1.65	Supplementary Fig. 7
8	$YAlO_3/KTaO_3$, Sample #5	780 °C	22.7	2.06	Supplementary Fig. 8
9	$LaAlO_3/KTaO_3$, Sample #6	600 °C	7.44	1.44	Supplementary Fig. 9
10	$YAlO_3/KTaO_3$, Sample #7	780 °C	7.1	1.6	Supplementary Fig. 10

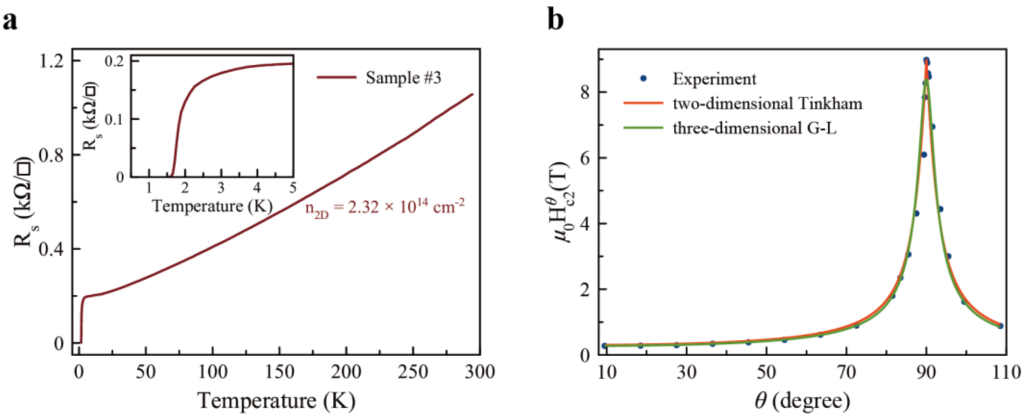

Supplementary Fig. 6| Electrical properties for a-$YAlO_3$/$KTaO_3$(111) (Sample #3). a Electrical resistance R_s as a function of temperature at zero magnetic field for a-$YAlO_3$/$KTaO_3$(111) with charge carrier density of 2.32×10^{14} cm^{-2} measured at 5 K (Sample #3 with growth temperature of 780 °C). Low temperature-dependent R_s is illustrated in the inset of (a). **b** Upper critical field $\mu_0 H_{c2}^{\theta}$ as a function of out-of-plane polar angle θ with a fixed temperature of 1.5 K. The red and green solid lines are fitted to the data using the two-dimensional Tinkham model and three-dimensional Ginzburg-Landau (G-L) model, respectively.

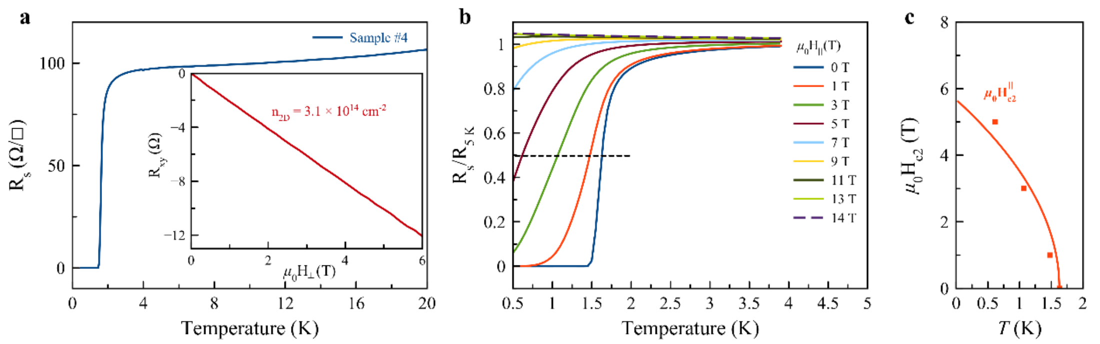

Supplementary Fig. 7| Electrical properties for a-$YAlO_3$/$KTaO_3$(111) (Sample #4). a Electrical resistance R_s as a function of temperature in the absence of magnetic field for a-$YAlO_3$/$KTaO_3$(111) with charge carrier density of 3.1×10^{14} cm^{-2} measured at 5 K (Sample #4 with growth temperature of 785 °C). The corresponding perpendicular field ($\mu_0H_\perp$)-dependent transverse Hall resistance R_{xy} measured at the temperature of 5 K is illustrated in the inset of (a). **b** Rescaled magnetoresistance R_s/R_{5K} for fields parallel to the plane surface of Sample #4. R_{5K} denotes the electrical resistance R_s measured at the temperature of 5 K in the absence of field. **c** Temperature dependence of the extracted upper critical field μ_0H_{c2} in (b) ($\mu_0H_{c2}^{\parallel}$ for the in-plane field along the $[11\bar{2}]$-axis shown in Fig. 1d).

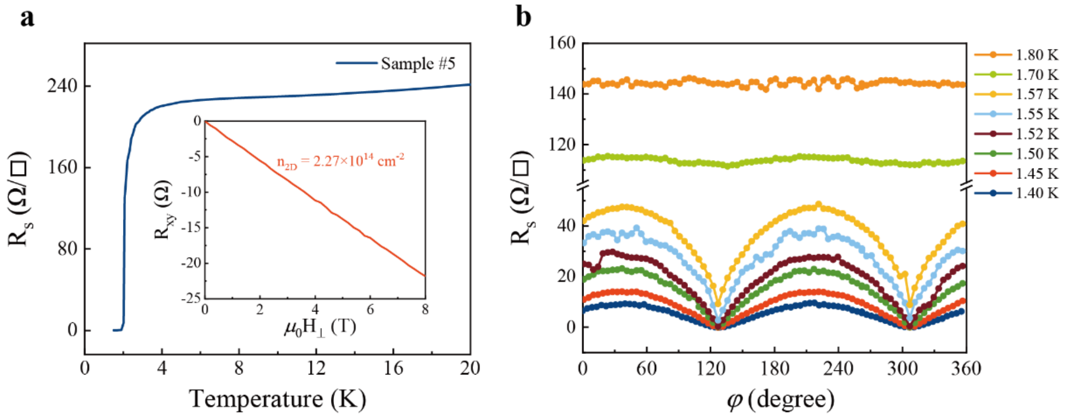

Supplementary Fig. 8| Twofold anisotropic property in the superconducting state in a-YAlO$_3$/KTaO$_3$(111) (Sample #5). a Electrical resistance R_s as a function of temperature in the absence of magnetic field for a-YAlO$_3$/KTaO$_3$(111) with charge carrier density of 2.27×10^{14} cm^{-2} measured at 5 K (Sample #5 with growth temperature of 780 °C). The corresponding perpendicular field ($\mu_0H_\perp$)-dependent transverse Hall resistance R_{xy} measured at the temperature of 5 K is illustrated in the inset of (a). **b** In-plane angular φ-dependent magnetoresistance R_s at various temperatures for an applied field of 1 T by using the vector magnet. The φ is defined as the in-plane azimuthal angle between the applied field and the $[1\bar{1}0]$-axis of the lattice shown in Fig. 1d. Here, the conspicuous signal of twofold symmetric magnetoresistance in the superconducting state has also been observed, providing further evidence for the intrinsic nature of the twofold anisotropic superconductivity at the KTaO$_3$ heterointerfaces.

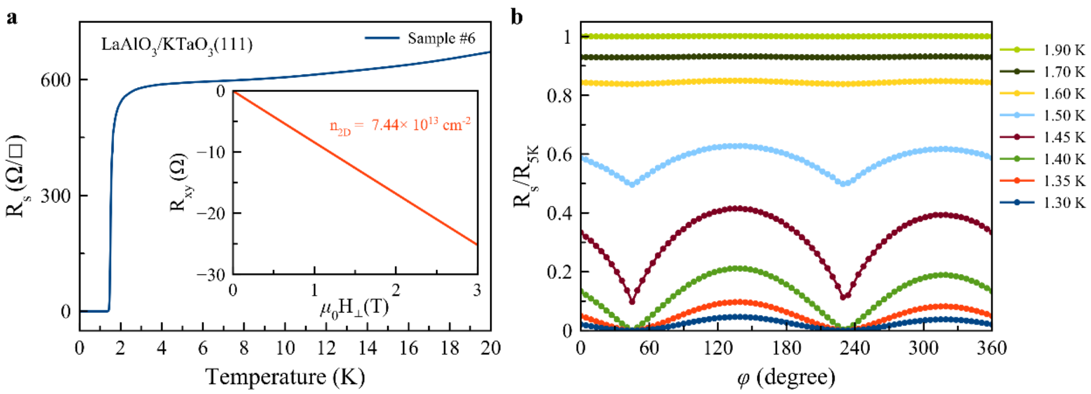

Supplementary Fig. 9| Twofold anisotropic property in the superconducting state in a-LaAlO$_3$/KTaO$_3$(111) (Sample #6). a Electrical resistance R_s as a function of temperature in the absence of magnetic field for a-LaAlO$_3$/KTaO$_3$(111) with charge carrier density of 7.44×10^{13} cm^{-2} measured at 5 K (Sample #6 with growth temperature of 600 °C). The corresponding perpendicular field ($\mu_0 H_\perp$)-dependent transverse Hall resistance R_{xy} measured at the temperature of 5 K is illustrated in the inset of (a). Here, the a-LaAlO$_3$ thin films are grown on the KTaO$_3$(111) substrates by pulsed laser deposition using the same growth conditions to that of a-YAlO$_3$ thin films. **b** In-plane angular φ-dependent rescaled magnetoresistance R_s/R_{5K} at various temperatures for an applied field of 1 T by using the vector magnet. R_{5K} denotes the electrical resistance R_s measured at the temperature of 5 K in the absence of field, and the φ is defined as the in-plane azimuthal angle between the applied field and the $[1\bar{1}0]$-axis of the lattice shown in Fig. 1d. Here, the conspicuous signal of twofold symmetric magnetoresistance in the superconducting state has also been observed, providing additional evidence for the intrinsic nature of the twofold anisotropic superconductivity at the KTaO$_3$ heterointerfaces.

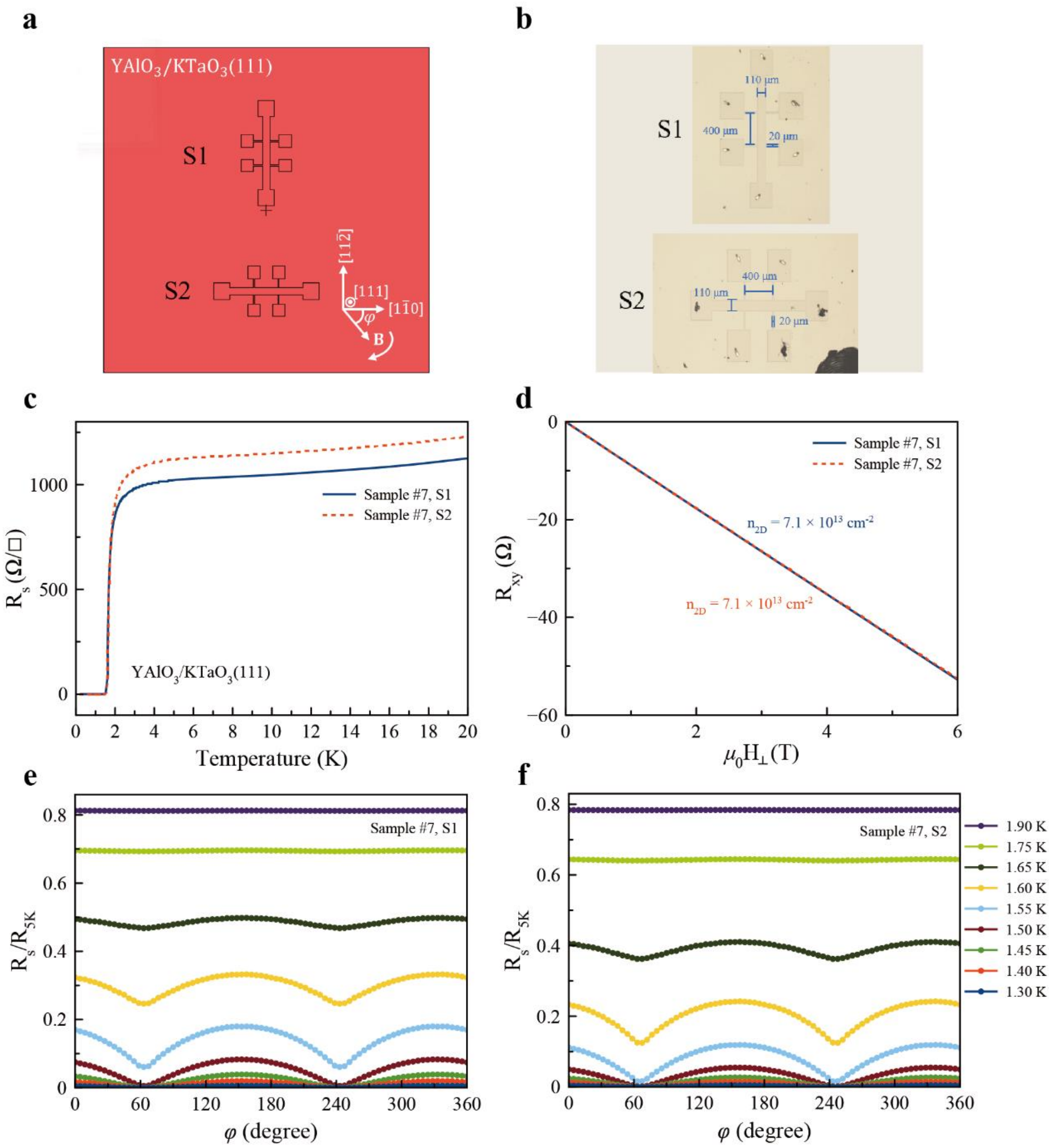

Supplementary Fig. 10| Crystalline orientation-independence of twofold anisotropic property in the superconducting state in a-$YAlO_3$/$KTaO_3$(111). a Schematic and **b** optical microscopic images of the fabricated two perpendicular Hall bar configurations of the a-$YAlO_3$/$KTaO_3$(111) heterointerfaces (Sample #7, S1 and S2, with growth temperature of 780 °C). The φ is defined as the in-plane azimuthal angle between the applied field and the $[1\bar{1}0]$-axis of the lattice shown in the inset of (a), and the precise geometry of the Hall bar configurations is documented in (b). **c** Electrical resistance R_s as a function of temperature in the absence of magnetic field for the two Hall bar devices of the a-$YAlO_3$/$KTaO_3$(111). **d** Corresponding perpendicular field ($\mu_0 H_\perp$)-dependent transverse Hall resistance R_{xy} measured at the temperature of 5 K. **e**,**f** In-plane angular φ-dependent rescaled magnetoresistance R_s/R_{5K} at various temperatures for an applied field of 1 T by using the vector magnet for the two Hall bar devices of the a-$YAlO_3$/$KTaO_3$(111). R_{5K} denotes the electrical resistance R_s measured at the temperature of 5 K in the absence of field. Here, the conspicuous signal of twofold symmetric magnetoresistance in the superconducting state for the two perpendicular Hall bar

devices has also been observed, providing further evidence for the intrinsic nature of the twofold anisotropic superconductivity at the $KTaO_3$ heterointerfaces.

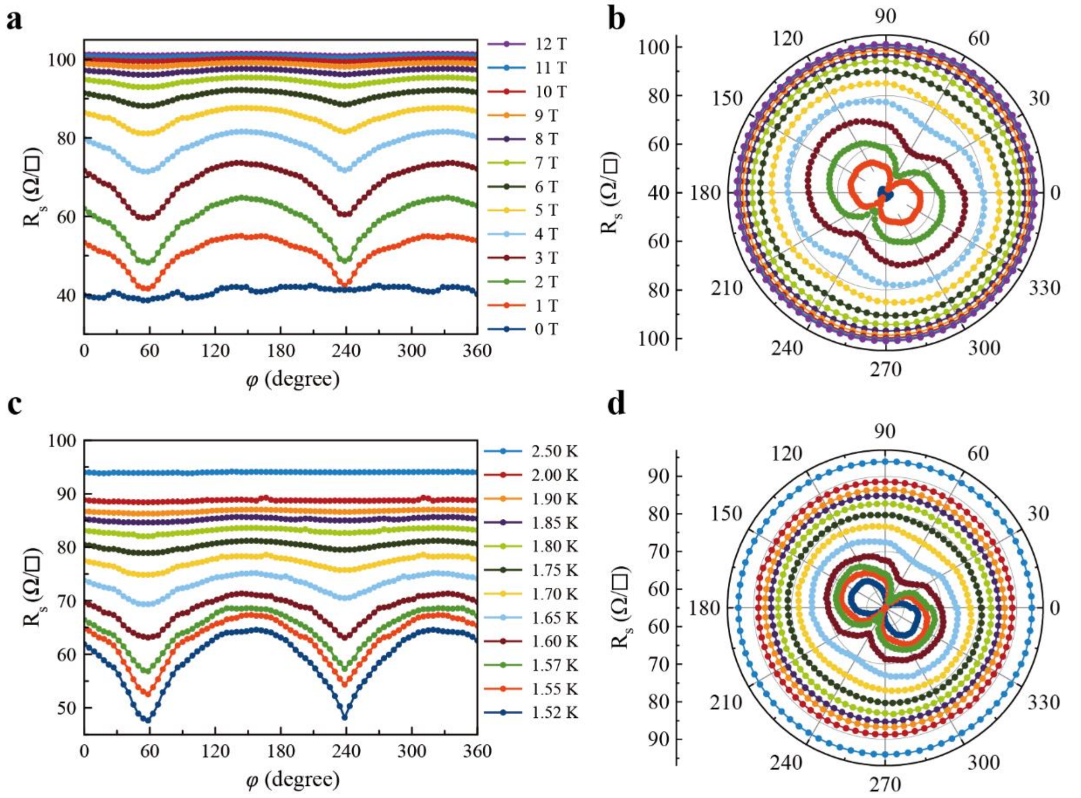

Supplementary Fig. 11| Magnetic field and temperature-dependent twofold anisotropic magnetoresistance in the superconducting state in a-$YAlO_3$/$KTaO_3$(111). a In-plane azimuthal angular φ-dependent magnetoresistance R_s as a function of an applied in-plane field at a fixed temperature of 1.52 K by using a mechanical rotator in a ^{4}He cryostat (Sample #4). The φ is defined as the in-plane azimuthal angle between the applied field and the $[1\bar{1}0]$-axis of the lattice shown in Fig. 1d. **b** Polar plots of the data in (a). **c** In-plane azimuthal angular φ-dependent magnetoresistance R_s as a function of temperature with a fixed field of 2 T by using a mechanical rotator in a ^{4}He cryostat (Sample #4). **d** Polar plots of the data in (c).

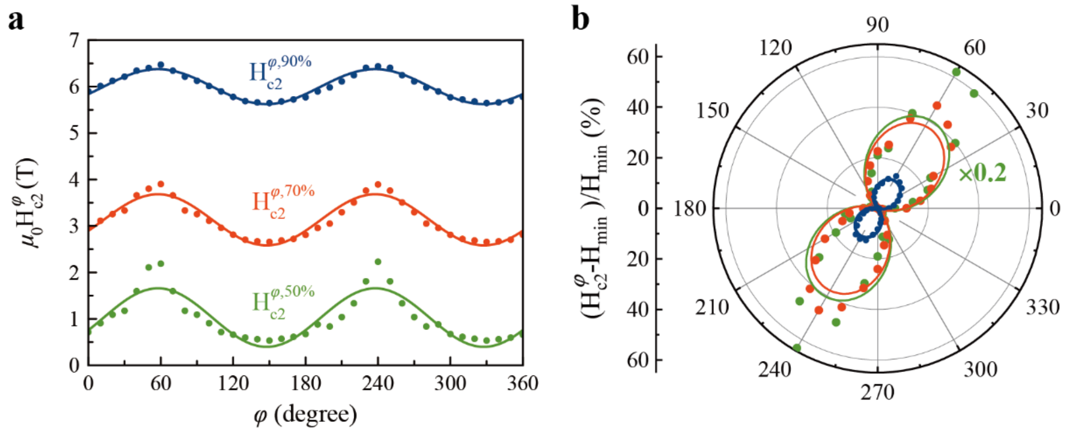

Supplementary Fig. 12| Criterion independence of determined H_{c2}^{φ}. a H_{c2}^{φ} determined from various criterion with 90 %, 70 %, and 50 % of the normal state resistance at a temperature of 5 K by using a mechanical rotator in a ^{4}He cryostat (Sample #4). The φ is defined as the in-plane azimuthal angle between the applied field and the $[1\bar{1}0]$-axis of the lattice shown in Fig. 1d. **b** Polar plot of the data normalized by the minimum value of H_{c2}^{φ} (H_{min}) in (a). Notably, the twofold rotational symmetry of H_{c2} qualitatively remains unaltered by changing different criteria.

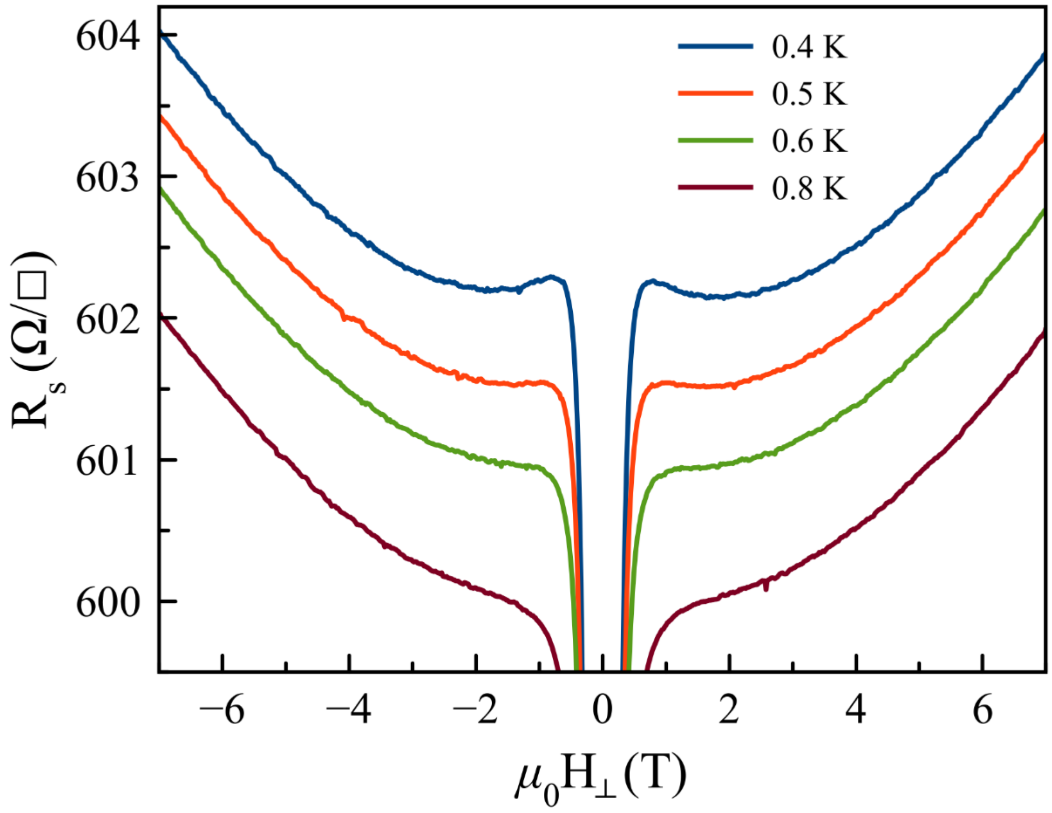

Supplementary Fig. 13| Magnetoresistance as a function of an applied out-of-plane field. Out-of-plane magnetic field-dependent magnetoresistance R_s at various temperatures on a-$YAlO_3/KTaO_3(111)$ (Sample #2). Here, it is interesting to point out that the appearance of negative magnetoresistance R_s (weak localization) is perceived at the verge of superconductivity with an application of out-of-plane magnetic field at various temperatures. Such a similar behavior has also been reported for the two-dimensional electron gases at the sister c-$LaAlO_3/SrTiO_3$ heterointerfaces and is interpreted as a consequence of Rashba spin-orbit coupling[SI8-SI11], suggesting the existence of strong spin-orbit coupling at the $KTaO_3$ heterointerfaces.

Supplementary Table 2. Character table for point group of C_{3v}.

C_{3v}	E	$2C_3$(z)	$3\sigma_v$	linear functions, rotations	quadratic functions	cubic functions
A_1	+1	+1	+1	z	x^2+y^2, z^2	z^3, $x(x^2-3y^2)$, $z(x^2+y^2)$
A_2	+1	+1	-1	R_z	-	$y(3x^2-y^2)$
E	+2	-1	0	(x, y), (R_x, R_y)	(x^2-y^2, xy), (xz, yz)	(xz^2, yz^2), $[xyz, z(x^2-y^2)]$, $[x(x^2+y^2), y(x^2+y^2)]$

SI References

SI1. Michaeli, K., Potter, A. C., & Lee, P. A. Superconducting and ferromagnetic phases in $SrTiO_3/LaAlO_3$ oxide interface structures: Possibility of finite momentum pairing. *Phys. Rev. Lett.* **108**, 117003 (2012).

SI2. Li, L. et al. Coexistence of magnetic order and two-dimensional superconductivity at $LaAlO_3/SrTiO_3$ interfaces. *Nat. Phys.* **7**, 762 (2011).

SI3. Bert, J. A. et al. Direct imaging of the coexistence of ferromagnetism and superconductivity at the $LaAlO_3/SrTiO_3$ interface. *Nat. Phys.* **7**, 767 (2011).

SI4. Dikin, D. A. et al. Coexistence of superconductivity and ferromagnetism in two dimensions. *Phys. Rev. Lett.* **107**, 056802 (2011).

SI5. Jiang, D. et al. Strong in-plane magnetic field-induced reemergent superconductivity in the van der Waals heterointerface of $NbSe_2$ and $CrCl_3$. *ACS Appl. Mater. Interfaces* **12**, 49252 (2020).

SI6. Pan, Y. et al. Rotational symmetry breaking in the topological superconductor $Sr_xBi_2Se_3$ probed by upper-critical field experiments. *Sci. Rep.* **6**, 28632 (2016).

SI7. Yonezawa, S. Nematic superconductivity in doped Bi_2Se_3 topological superconductors. *Condens. Matter* **4**, 2 (2019).

SI8. Diez, M. et al. Giant negative magnetoresistance driven by spin-orbit coupling at the $LaAlO_3/SrTiO_3$ interface. *Phys. Rev. Lett.* **115**, 016803 (2015).

SI9. Joshua, A. et al. A universal critical density underlying the physics of electrons at the $LaAlO_3/SrTiO_3$ interface. *Nat. Commun.* **3**, 1129 (2012).

SI10. Yang, M. et al. High field magneto-transport in two-dimensional electron gas $LaAlO_3/SrTiO_3$. *Appl. Phys. Lett.* **109**, 122106 (2016).

SI11. Caviglia, A. D. et al. Tunable Rashba spin-orbit interaction at oxide interfaces. *Phys. Rev. Lett.* **104**, 126803 (2010).
